# Subtle Influence of ACE2 Glycan Processing on SARS-CoV-2 Recognition

**DOI:** 10.1016/j.jmb.2020.166762

**Published:** 2021-02-19

**Authors:** Joel D. Allen, Yasunori Watanabe, Himanshi Chawla, Maddy L. Newby, Max Crispin

**Affiliations:** 1 School of Biological Sciences, University of Southampton, Southampton SO17 1BJ, UK; 2 Oxford Glycobiology Institute, Department of Biochemistry, University of Oxford, South Parks Road, Oxford OX1 3QU, UK; 3 Division of Structural Biology, University of Oxford, Wellcome Centre for Human Genetics, Oxford OX3 7BN, UK

**Keywords:** SARS-CoV-2, ACE2, glycosylation, surface plasmon resonance, glycan engineering

## Abstract

The severity of SARS-CoV-2 infection is highly variable and yet the molecular basis for this effect remains elusive. One potential contribution are differences in the glycosylation of target human cells, particularly as SARS-CoV-2 has the capacity to bind sialic acid which is a common, and highly variable, terminal modification of glycans. The viral spike glycoprotein (S) of SARS-CoV-2 and the human cellular receptor, angiotensin-converting enzyme 2 (ACE2) are both densely glycosylated. We therefore sought to investigate whether the glycosylation state of ACE2 impacts the interaction with SARS-CoV-2 viral spike. We generated a panel of engineered ACE2 glycoforms which were analyzed by mass spectrometry to reveal the site-specific glycan modifications. We then probed the impact of ACE2 glycosylation on S binding and revealed a subtle sensitivity with hypersialylated or oligomannose-type glycans slightly impeding the interaction. In contrast, deglycosylation of ACE2 did not influence SARS-CoV-2 binding. Overall, ACE2 glycosylation does not significantly influence viral spike binding. We suggest that any role of glycosylation in the pathobiology of SARS-CoV-2 will lie beyond its immediate impact of receptor glycosylation on virus binding.

## Highlights

• N-linked glycans of ACE2 have been suggested to play a role in SARS-CoV-2 binding.
• Using glycan engineering we generated a panel of glycan modified ACE2 variants.
• The binding of these variants to spike protein was determined using SPR and LC-MS.
• These results suggest a limited role for the glycans of ACE2 in SARS-CoV-2 binding.
• SARS binding with ACE2 is slightly influenced by sialylation and mannosylation.

## Graphical abstract

**Figure:**
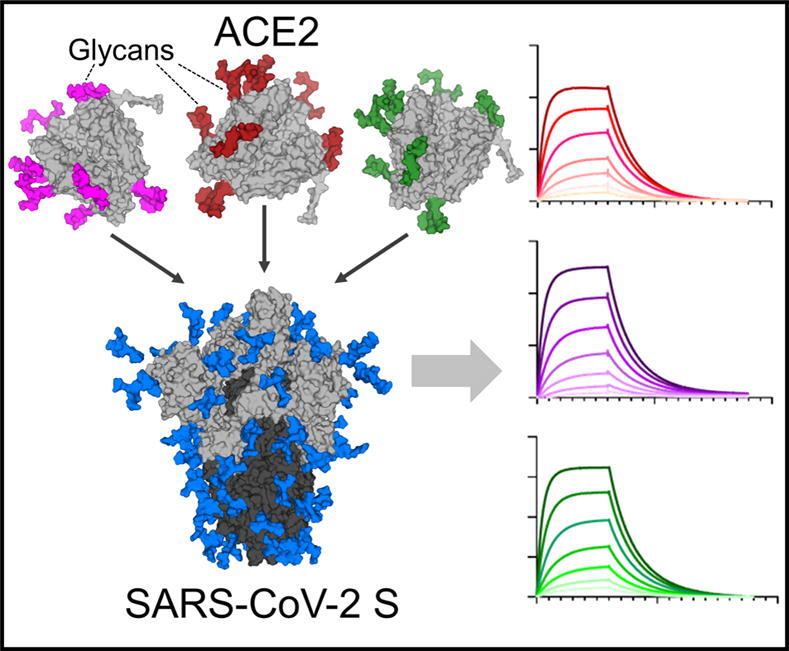

## Introduction

Severe acute respiratory syndrome coronavirus-2 (SARS-CoV-2), the causative agent of COVID-19, encodes an extensively glycosylated spike (S) protein that protrudes from the viral surface.^1^, ^2^ The S protein is a trimeric class I fusion protein, composed of two functional subunits, S1 and S2, which are responsible for receptor binding and membrane fusion, respectively.^1^, ^3^ The S protein binds to cell surface angiotensin-converting enzyme 2 (ACE2) which initiates host-cell entry and results in membrane fusion.^4^ Human ACE2 is a dimeric membrane-bound glycoprotein that encodes seven N-linked glycosylation sequons per protomer (UniProt KB: Q9BYF1). ACE2 glycosylation is likely to contribute to protein folding and stability,^5^, ^6^ and evidence is emerging that it can influence SARS-CoV-2 recognition.^7^, ^8^

High resolution structures have been published of the SARS-CoV-2-ACE2 complex, however, one limitation of these structures is the difficulty resolving the contribution of glycan-glycan and glycan-protein interactions.^9^, ^10^ It is also known that glycans can influence the binding between two macromolecules even when glycans are attached outside of the protein–protein interface^11^, ^12^, ^13^ and there is precedent for glycosylation of the host-receptor influencing viral binding interactions.^14^ In the case of the SARS-CoV-2 interaction, removal of the ACE2 glycan at N90 enhances binding to the receptor binding domain (RBD) of the S protein and it has been suggested that the processing state of this glycan may influence the interaction.^15^ The potential influence of ACE2 glycosylation on this interaction with the S protein has been further supported by molecular dynamics of the fully glycosylated complex.^16^, ^17^ It has been suggested that glycans may play a role in receptor binding as contacts are predicted between the glycans of ACE2 and both the protein and glycan components of the S protein.^16^, ^17^, ^18^ For example, the glycan at N546 of the ACE2 receptor is suggested to interact with the glycans at N74 and N165 on the S protein.^16^ Furthermore, the glycans of ACE2 at N90, N322 and N546 are all reported to interact with the protein moiety of the S protein.^16^ In contrast, O-glycosylation abundance is low, with the exception of T730. However, this site is distal to the binding interface and likely does not contribute to binding.^19^ Understanding the extent to which the glycan compositions, and the presence of the glycan itself, influence the binding of SARS-CoV-2 is important for both potential ACE2 therapeutic design and understanding the influence of host glycosylation on viral cell entry.

Beyond the steric effects of glycosylation on protein interactions, the binding capacity of viral glycoproteins directly towards carbohydrates can also be an important part of viral pathobiology. Indeed, the phenomenon of glycans on host-glycoproteins influencing and directly facilitating viral infection has been characterized on numerous viruses,^20^, ^21^ with perhaps the most well-known example being the sialic-acid binding capabilities of hemagglutinin of influenza A viruses.^20^ Importantly, a number of human coronaviruses have been shown to be able to bind sialosides, including Middle East respiratory syndrome (MERS)-CoV,^22^ SARS-CoV-1,^23^ HKU1^24^ and SARS-CoV-2.^25^, ^26^, ^27^ The ability of SARS-CoV-2 to bind charged carbohydrates is further supported by the observation that sulfated glycosaminoglycans bind to the viral spike glycoprotein and can inhibit infection.^28^, ^29^, ^30^, ^31^, ^32^ With the capacity of SARS-CoV-2 to directly recognize sialylated glycans it provides further impetus to explore the impact of ACE2 glycosylation on the binding to the S protein.

Here, we have modified the glycan structures on ACE2 using several glycan engineering techniques, which incorporated the use of glycosyltransferases, glycosidases and enzymatic inhibitors. Using a mass spectrometric approach, we determined the site-specific glycan structures present on ACE2 both with and without glycan engineering. Binding studies of the glycan variants of ACE2 were performed using surface plasmon resonance (SPR) to determine binding affinities between SARS-CoV-2 S and ACE2 variants. We reveal that when ACE2 glycans are hypersialylated, or when all glycans were converted to oligomannose-type, there was a modest decrease in affinity. When the sialic acid residues were removed a statistically significant but modest increase in affinity was observed. However, overall, the effects were subtle and deglycosylation had minimal impact on S binding.

## Results and Discussion

### Expression and purification of ACE2

**Figure 1:**
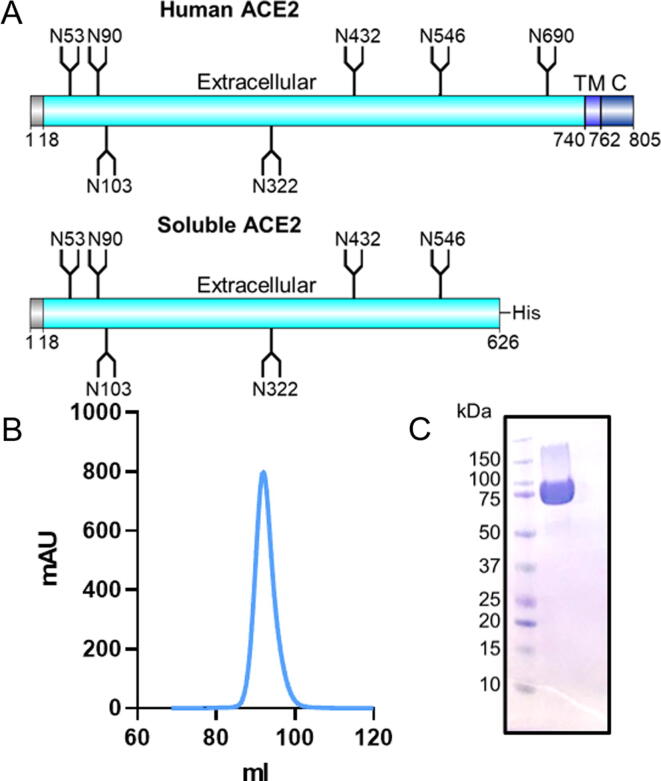
Production and purification of soluble ACE2. (A) Schematic of ACE2 comparing full length human ACE2 with the soluble ACE2 construct used in this study shown underneath. The positions of N-linked glycan sequons (N-X-S/T, where X ≠ P) are shown as forked sticks. (B) Size exclusion chromatogram of soluble ACE2 following nickel affinity purification. (C) SDS-PAGE using Coomassie stain of the pooled SEC fractions for soluble ACE2.

To enable the determination of the glycosylation and binding capabilities of ACE2, a soluble recombinant ACE2 ectodomain that possesses all bar the last N-linked glycan site was expressed in HEK 293F cells (Figure 1(A)). This monomeric material has previously been shown to bind S protein and has been structurally characterized by cryo-electron microscopy.^1^ Expressed material was purified by nickel-affinity chromatography and size exclusion chromatography (SEC). The SEC chromatogram demonstrated a single peak (Figure 1(B)) which exhibited a mass of ~75 kDa when subjected to SDS-PAGE analysis (Figure 1(C)). The soluble ACE2 expressed at ~80 mg/mL and ensured that all experiments performed were undertaken on the same batch of ACE2. To assay ACE2 binding we also expressed SARS-CoV-2 S protein which was expressed and purified in an identical manner to ACE2, as reported previously.^1^, ^2^

### ACE2 glycosylation analysis and SARS-CoV-2 S binding

**Figure 2:**
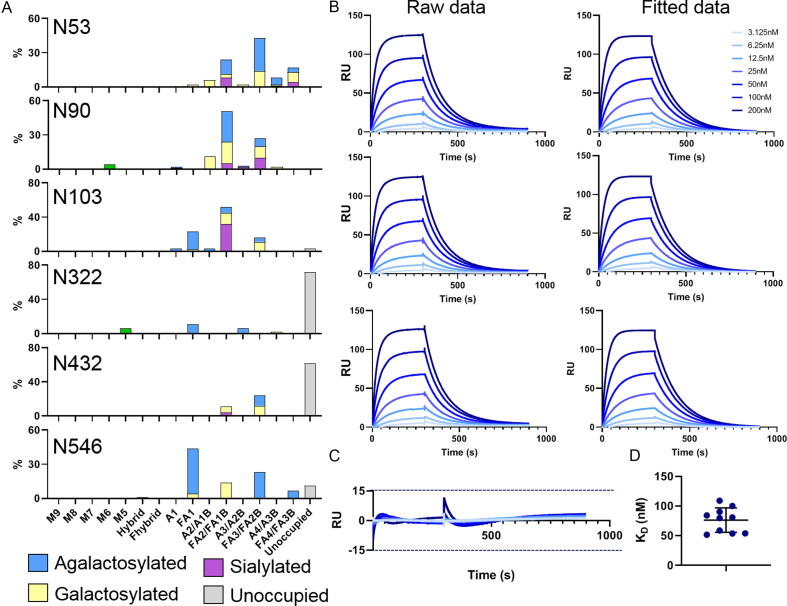
Site-specific glycan analysis and determination of SARS-CoV-2 binding of soluble ACE2. (A) Site-specific glycan analysis of soluble ACE2 determined by LC-MS. Glycans are categorized into oligomannose-type glycans (green) and complex-type glycans which are grouped according to the presence/absence of fucosylation as well as the number of processed antennae. Within these categories glycans are colored according to the presence of galactose (yellow), sialic acid (purple), and the absence of both (blue). The proportion of N-glycan sites which do not have a glycan attached are colored grey. Nomenclature: M5-M9, Man_5-9_GlcNAc_2_; F, fucose; A, antennae; B, bisecting GlcNAc. (B) Surface plasmon resonance sensorgrams for soluble ACE2 binding to SARS-CoV-2 S protein. One of the ten soluble ACE2 SPR experiments is shown. Each sensorgram represents one analytical repeat. The fitted were generated from the three repeats using Biacore Evaluation software with a 1:1 binding model. (C) Residuals comparing the deviation of the fitted data to the experimental data. (D) Plot of the *K*_D_ determined for each experimental repeat of soluble ACE2 binding to SARS-CoV-2 S protein. The mean is plotted as a black line and the error bars represent +/- standard deviation as calculated using GraphPad Prism.

To understand the impact of the ACE2 glycosylation upon SARS-CoV-2 binding we first defined the glycans present on the soluble variant used in this study (Figure 1(A)). Human ACE2 encodes seven N-linked glycosylation sites at N53, N90, N103, N322, N432, N546 and N690. The soluble construct used here lacks the N690 glycan, however, this glycosylation site lies distal to the protein interface with the S protein and is therefore not predicted to directly influence the interaction.^16^ To resolve the site-specific glycosylation, we employed trypsin, chymotrypsin, and alpha-lytic protease to generate glycopeptides, which were subsequently subjected to liquid-chromatography-mass spectrometry (LC-MS) analysis (Figure 2(A)). We have previously used this method to determine the site-specific glycosylation of a range of coronaviruses.^2^, ^33^ This analysis revealed a high abundance of complex-type glycans on ACE2, as would be expected for a mature, secreted mammalian glycoprotein. These observations are similar to those previously reported for various recombinant ACE2 glycoproteins.^16^, ^19^ Interestingly, whilst the glycan sites at N53, N90, N103, and N546 were occupied with complex-type glycans, N322 and N432 exhibited low levels of glycan site occupancy with the unmodified peptide accounting for 72% and 62% for each respective site. This underoccupancy has been observed in another analysis of ACE2 glycoproteins.^16^ We hypothesize that the extent of under occupancy may exceed that of native, full-length ACE2 and be driven, to some extent, by the high expression levels of ACE2 in the recombinant system. This phenomenon has been observed for HIV-1 where soluble immunogens exhibit lower glycan occupancy compared to full-length, membrane bound, material.^34^, ^35^

As we have applied an LC-MS methodology analyzing glycopeptides it is therefore possible to categorize the heterogenous glycan populations according to their terminal monosaccharides (Figure 2(A)). Glycans were grouped according to the number of processed antennae, the presence or absence of fucose, the presence or absence of galactose and if the glycan contains sialic acid. The predominant type of glycan observed across all five sites were fucosylated bi- and tri-antennary complex-type glycans which lack galactose and sialic acid. The exception to this is at N103 where a high abundance of sialylated glycans was observed. Overall high levels of fucosylation were observed across all glycans (66%; Figure S1). Galactosylation was moderately abundant (37%) and sialylated N-glycans were comparably low (15%). This pattern of high fucosylation, moderate galactosylation and low sialylation is typical of protein expressed using recombinant cell lines such as HEK 293F.^34^, ^36^, ^37^

To understand the impact of different glycans upon ACE2 binding we initially investigated the interaction between the un-engineered soluble ACE2 (referred to here as the wild-type form, WT) and SARS-CoV-2 S glycoprotein. We used SPR to define the dissociation constant (*K*_D_). Prior to analysis the His-Tag at the C-terminus of ACE2 was removed using HRV3C protease to minimize ACE2 binding to the sensor chip. For each analysis three repeats were performed with serial dilutions of ACE2 ranging from 200 nM to 3.125 nM (Figure 2(B)). To calculate the binding parameters a fitted curve was generated using a 1:1 binding model. The sensorgrams showed minimal deviation when compared to the fitted curves (Figure 2(C)). When calculating binding kinetics, the average of three analytical repeats was used. To understand the batch-to-batch variation in SARS-CoV-2 affinity for ACE2, we performed a series of experiments with different batches of SARS-CoV-2 glycoprotein with a single ACE2 control in each in which no glycan engineering had been performed. In total, 10 ACE2 sensorgrams were obtained. The measured *K*_D_ for the interaction between SARS-CoV-2 and ACE2 varied between 50 and 100 nM with an average of 76 nM (Figure 2(D)).

### ACE2 glycosylation modestly impacts binding to SARS-CoV-2

**Figure 3:**
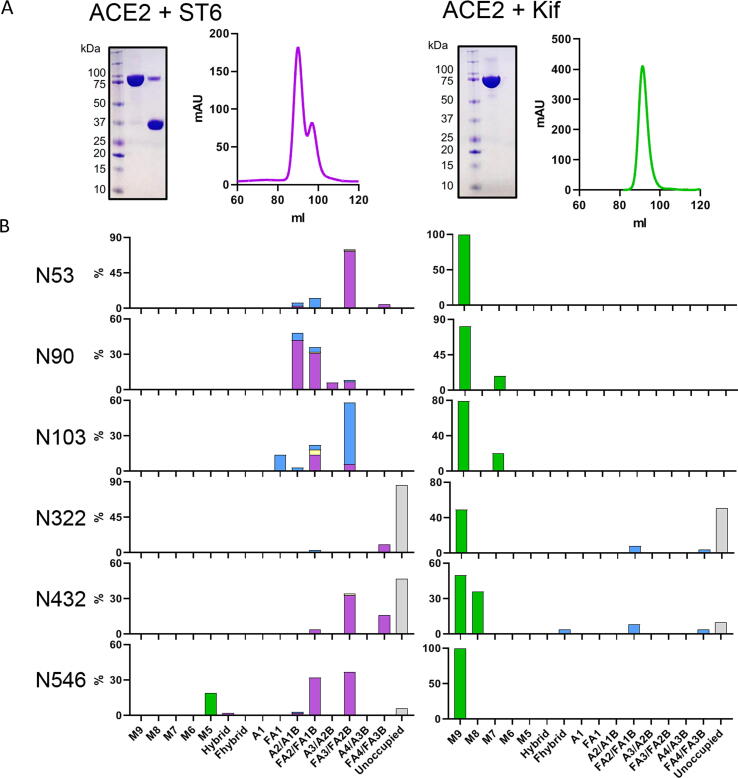
Glycan modification of soluble ACE2 using ST6 and kifunensine co-expression. (A) SDS-PAGE and SEC trace for ACE2 co-expressed with ST6 and also ACE2 expressed with 20 µM kifunensine. The two peaks shown in SEC were pooled separately and show two distinct bands corresponding to soluble ACE2 (~75 kDa) and ST6 (~37 kDa). (B) Site-specific glycan analysis of ACE2 expressed with ST6 and ACE2 expressed with kifunensine. Bars are categorized and colored according to Figure 2(A).

In order to probe the influence of different glycoforms of ACE2 on SARS-CoV-2 S binding, we generated glycan modified versions of the receptor. We employed a range of different approaches including modifying the glycans during expression of ACE2 and using glycosidases post-expression to remove specific monosaccharides. To investigate the impact of sialylation upon ACE2 binding, we co-expressed ACE2 with a solubilized variant of β-galactoside α-2,6-sialyltransferase I (ST6).^38^ As the ST6 contains a His-Tag, the SEC contained two peaks which, when analyzed by SDS-PAGE, confirmed two proteins, one at ~75 kDa and one at 35 kDa, corresponding to ACE2 and ST6 respectively (Figure 3(A)). To determine whether ST6 co-expression increased sialylation we employed the same LC-MS approach outlined for WT ACE2. This demonstrated a notable increase in sialylation at every site, with the exception of N103 which exhibits a high degree of sialylation on the WT protein and was not enhanced by ST6 co-expression (Figure 3(B)). The total sialylation increased from 15% for WT ACE2 to 54% for ACE2 co-expressed with ST6 (Supplementary Table 2). The levels of fucosylation and unoccupancy were comparable to the WT ACE2.

In addition to sialylation, we investigated the impact of artificially adding oligomannose-type glycans at each site across ACE2. These glycans are rare on healthy host glycoproteins and it is unlikely these glycans are in high abundance on cellular ACE2 although oligomannose-type glycans can arise naturally on cellular receptors depending on the producer cell.^39^, ^40^ To convert the glycans to predominantly Man_9_GlcNAc_2_, 20 µM of kifunensine (Kif) was added at the time of transfection. This enzyme inhibits the glycan-processing enzyme, ER α-mannosidase I, which results in the presentation of oligomannose glycans on the mature glycoprotein. The resultant ACE2, which we term here Kif ACE2, was purified and, as for WT ACE2, a single peak was observed by SEC (Figure 3(A)). Glycan analysis confirmed inhibition of glycan processing, with large populations of oligomannose-type glycans at every site (Figure 3(B)).

To further modify the glycans of ACE2 we used the three glycan variants as a platform to generate several glycan modified variants of ACE2. For each approach LC-MS was performed to confirm successful enzymatic digestion of the glycans (Supplementary Table 3). To assay whether the presence of a glycan impacts ACE2 binding we removed the oligomannose-type glycans from Kif ACE2 using endoglycosidase H (endoH) which leaves a single N-acetylglucosamine residue.^41^ This approach was taken as opposed to removing all the glycans from WT ACE2 as the total removal of N-glycans from proteins can cause misfolding, whereas the contribution of a single N-acetylglucosamine to ACE2 binding will likely be minimal. This approach is commonly utilized to obtain crystal structures of deglycosylated proteins while avoiding the complications of misfolding and incomplete digestion that can be associated with the use of peptide N-glycosidase.^41^ To further understand the impact of sialylation, we removed the sialic acids from both WT and ST6 ACE2 using α2-3,6,8 sialidase. The role of fucose was investigated using WT ACE2 treated with α-fucosidase. Each of these proteins were characterized by LC-MS and used for subsequent analysis by SPR. Overall, we demonstrated that it is possible to modulate the glycosylation of ACE2 in a specific manner for downstream analyses.

**Figure 4:**
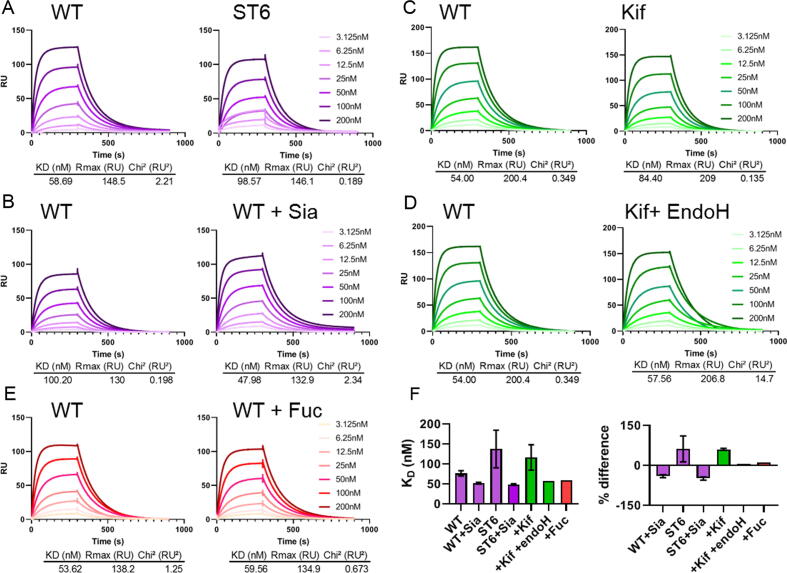
SPR analysis of glycan modified soluble ACE2 binding to SARS-CoV-2 S protein. (A) Comparison of sensorgrams for WT ACE2 and ACE2 co-expressed with ST6. Both sensorgrams were obtained using the same SARS-CoV-2 S protein preparation, each WT ACE2 experiment detailed was performed using the same batch as the corresponding glycan modified ACE2. All displayed curves represent the average of three analytical repeats. All kinetic parameters were calculated using a 1:1 binding model using the Biacore evaluation software. (B) Sensorgrams comparing WT soluble ACE2 and WT ACE2 treated with sialidase. (C) Sensorgrams comparing WT ACE2 and ACE2 expressed in the presence of 20 µM kifunensine. (D) Sensorgrams comparing WT ACE2 and ACE2 expressed in the presence of 20 µM kifunensine followed by enzymatic digestion with endoH. (E) Sensorgrams comparing WT ACE2 with WT ACE2 enzymatically digested with fucosidase. (F) Calculated *K*_D_ for each of the ACE2 variants used, including repeats. Comparison of the % difference in *K*_D_ for each glycan modified ACE2 variant. Percentage difference was calculated for each glycan modified variant compared to a WT ACE2 sensorgram performed during the same SPR experiment. The average of each repeat of this analysis is shown, with error bars representing ±SEM shown where appropriate. ST6 ACE2, WT Sia ACE2, ST6 + ACE2 and Kif ACE2 were performed in triplicate whereas Kif + endoH and ACE2 + Fuc were performed once.

Using an identical experimental set up as for WT ACE2, we performed a series of experiments using both WT ACE2 and glycan modified ACE2. When ACE2 was co-expressed with ST6 a modest increase in *K*_D_ was observed, which corresponds to a decrease in binding (Figure 4(A)). When terminal sialic acid residues are removed using sialidase from both WT and hypersialylated ACE2 the affinity does not revert to WT but instead converges to a *K*_D_ ~ 30% lower, indicating that ACE2 treated with sialidase has a higher affinity for SARS-CoV-2 compared to WT ACE2 (Figure 4(B), (F)). The observation that the viral S protein interaction with ACE2 is only slightly influenced by ACE2 sialylation suggest that this interaction is robust to variations in sialylation state. Similarly, the presence or absence of fucose does not impact ACE2 binding to a significant extent (Figure 4(E)).

When all N-linked glycans on ACE2 are converted to oligomannose-type glycans using kifunensine the *K*_D_ increases by ~50%, corresponding to a decrease in binding (Figure 4(C)). However, when these oligomannose-type glycans are removed by endoH, leaving only a single N-acetylglucosamine residue remains, the affinities are comparable with WT ACE2 (Figure 4(D)). However, the impact of mannosylation suggest that viral S protein more optimally binds ACE2 glycoprotein that has undergone glycan maturation during biogenesis.

These effects are subtle, and it is important observation that deglycosylated ACE2 is capable of binding SARS-CoV-2 S protein (Figure 4(D)). To confirm our initial observations regarding the binding of glycan modified ACE2 to SARS-CoV-2 we reproduced the results and confirmed the initial observations. These experiments confirmed the significant difference between untreated ACE2 and sialidase treated ACE2 (*p* = 0.04) and also ST6 ACE2 treated with sialidase (*p* = 0.03) (Figure 4(F) and Supplementary Table 4) but also highlighted the subtlety of the observed effects as no other observed differences were significant. There was extensive variability between ST6 ACE2 experiments and the observed differences in *K*_D_ were not statistically significant. This could be due to the impact of microheterogeneity amongst hypersialylated ACE2 glycoforms leading to elevation in variability when compared to other samples.

### Perspectives

In this study, we probed the impact of variable glycosylation on ACE2 and SARS-CoV-2 S interaction. By modifying the glycans using glycosyltransferases, glycosidases, and inhibitors of the glycan-processing pathway, we engineered glycan variants of soluble ACE2 and performed binding studies with trimeric SARS-CoV-2 S. We reveal that, whilst the impact of fucosylation and the removal of the N-linked glycans was minimal, the removal and addition of sialic acids on ACE2 resulted in the increase and decrease of ACE2/SARS-CoV-2 S binding affinities, respectively. It was also interesting to note that kifunensine-treated ACE2 resulted in a decrease in binding affinity, which was recovered to the WT binding affinity when all but the first GlcNAc was removed by endoH. This indicates that there may be unfavorable interactions when the carbohydrate modifications on ACE2 are predominantly bulky Man_9_GlcNAc_2_ glycans.

These results suggest a limited role for the glycans of ACE2 in SARS-CoV-2 binding. When a glycan is critical for binding, the removal of the entire glycan will negatively impact binding.^42^ Likewise when a glycan is shielding an antigenic protein surface the removal of the entire glycan can increase the affinity to its binding partner.^43^ In this case, the removal of glycans from ACE2 has minimal effects on SARS-CoV-2 binding, revealing that the glycans do not play a critical role in this interaction. What these studies suggest, however, is that the binding of ACE2 to SARS-CoV-2 S protein can be influenced by the particular composition of ACE2 glycans, albeit only to a modest degree. We note that there is growing evidence for a role in sialic acid binding of SARS-CoV-2 on the cell surface, however this data suggests that the binding pocket for sialic acid is not productively utilized by ACE2 receptor glycans.^25^, ^26^, ^27^, ^44^

The use of recombinant ACE2 is being developed as a therapeutic against COVID-19^15^, ^45^, ^46^, ^47^, ^48^ and our results suggest that the SARS-CoV-2 S protein can bind to ACE2 promiscuously regardless of its glycosylation status. Whilst we do not know the impact of glycosylation *in vivo* or in a clinical setting our data suggests that therapeutic manufacture may be facilitated by a lack of strict glycan dependencies in its mode of recognition. However, we recognize that sialylation of therapeutics can influence their half-life.^49^ Overall, although ACE2 receptor glycosylation modestly impacts viral spike binding, it is highly likely that the glycosylation of virus and host target cells will have a significant impact upon the pathobiology of COVID-19 due to the extensive range of host lectins and the capacity of SARS-CoV-2 S protein to interact with host glycans. We conclude that any role of glycosylation in the pathobiology of SARS-CoV-2 will lie beyond its immediate impact of receptor glycosylation on virus binding.

## Methods

### Expression and purification of trimeric SARS-CoV-2 spike

To express the prefusion S ectodomain, a gene encoding residues 1–1208 of SARS-CoV-2 S (GenBank: MN908947) with proline substitutions at residues 986 and 987, a “GSAS” substitution at the furin cleavage site (residues 682–685), a C-terminal T4 fibritin trimerization motif, an HRV3C protease cleavage site, a TwinStrepTag and an 8XHisTag was synthesized and cloned into the mammalian expression vector pαH. Expression plasmid encoding SARS-CoV-2 S glycoprotein was transiently transfected into Human Embryonic Kidney (HEK) 293F cells. Cells were maintained at a density of 0.2–3 × 10^6^ cells per ml at 37 °C, 8% CO_2_ and 125 rpm shaking in FreeStyle 293F media (Fisher Scientific). Prior to transfection two solutions containing 25 mL Opti-MEM (Fisher Scientific) medium were prepared. Plasmid DNA was added to one to give a final concentration after transfection of 310 μg/L. Polyethylenimine (PEI) max reagent (1 mg/mL, pH 7) was added to the second solution to give a ratio of 3:1 PEI max: plasmid DNA. The two solutions were combined and incubated for 30 minutes at room temperature. Cells were transfected at a density of 1 × 10^6^ cells per mL and incubated for 7 days at 37 °C with 8% CO_2_ and 125 rpm shaking.

After harvesting, the cells were spun down at 4000 rpm for 30 minutes and the supernatant applied to a 500 mL Stericup-HV sterile vacuum filtration system (Merck) with a pore size of 0.22 µm. The supernatant containing SARS-CoV-2 S protein was purified using 5 mL HisTrap FF column connected to an Akta Pure system (GE Healthcare). Prior to loading the sample, the column was washed with 10 column volumes of washing buffer (50 mM Na_2_PO_4_, 300 mM NaCl) at pH 7. The sample was loaded onto the column at a speed of 2 mL/min. The column was washed with washing buffer (10 column volumes) containing 50 mM imidazole and eluted in 3 column volumes of elution buffer (300 mM imidazole in washing buffer). The elution was concentrated by a Vivaspin column (100 kDa cut-off) to a volume of 1 mL and buffer exchanged to phosphate buffered saline (PBS).

The Superdex 200 16 600 column was washed with PBS at a rate of 1 mL/min. After 2 hours, 1 mL of the nickel affinity purified material was injected into the column. Fractions separated by SEC were pooled according to their corresponding peaks on the Size Exclusion chromatograms. The target fraction was concentrated in 100 kDa vivaspin (GE healthcare) tubes to ~1 mL.

### Expression and purification of ACE2

FreeStyle293F cells (Thermo Fisher) were transfected with polyetyhlenimine and a plasmid encoding residues 1–626 of human ACE2 with a C-terminal HRV3C protease cleavage site, a TwinStrep Tag and an 8 × HisTag. This construct is identical to full length ACE2 except is truncated at position 626. This protein was expressed and purified identically as for the SARS-CoV2 glycoprotein, with the exception of a smaller Vivaspin cutoff being used for buffer exchanging. To produce oligomannose-type glycans on ACE2 20 μM kifunensine was added at the time of transfection. For ST6 coexpression DNA plasmid encoding ST6 was cotransfected with plasmid encoding ACE2 at a ratio of 2:1.

### Glycosidase digests

To generate glycan modified ACE2 separate aliquots of ACE2 were digested with a range of glycosidases: α1-2,3,4,6 fucosidase, α2-3, 6, 8 sialidase and endoglycosidase H (NEB). Glycosidases were added at a ratio of 1:20 and incubated at 37 °C overnight.

### His-Tag removal of ACE2

Following purification, the His-Tag was removed from ACE2 using HRV3C protease cleavage (Thermo Fisher). Digestion was performed at a ratio of 1:20 HRV3C protease: ACE2 in 1 × HRV3C reaction buffer (Thermo Fisher) and incubated at 4 °C overnight. To remove the HRV3C and uncleaved ACE2 nickel affinity chromatography was performed, except the flow through was collected rather than the elution.

### Mass spectrometry of glycopeptides

30 μg aliquots of ACE2 and ACE2 glycan variants were denatured for 1 h in 50 mM Tris/HCl, pH 8.0 containing 6 M of urea and 5 mM dithiothreitol (DTT). Next, the proteins were reduced and alkylated by adding 20 mM iodoacetamide (IAA) and incubated for 1 h in the dark, followed by a 1 h incubation with 20 mM DTT to eliminate residual IAA. The alkylated proteins were buffer-exchanged into 50 mM Tris/HCl, pH 8.0 using Vivaspin columns (3 kDa) and digested separately overnight using trypsin, chymotrypsin or alpha lytic protease (Mass Spectrometry Grade, Promega) at a ratio of 1:30 (w/w). The next day, the peptides were dried and extracted using C18 Zip-tip (MerckMilipore). The peptides were dried again, re-suspended in 0.1% formic acid and analyzed by nanoLC-ESI MS with an Easy-nLC 1200 (Thermo Fisher Scientific) system coupled to a Fusion mass spectrometer (Thermo Fisher Scientific) using higher energy collision-induced dissociation (HCD) fragmentation. Peptides were separated using an EasySpray PepMap RSLC C18 column (75 µm × 75 cm). A trapping column (PepMap 100 C18 3 μm (particle size), 75 μm × 2 cm) was used in line with the LC prior to separation with the analytical column. The LC conditions were as follows: 275 minute linear gradient consisting of 0–32% acetonitrile in 0.1% formic acid over 240 minutes followed by 35 minutes of 80% acetonitrile in 0.1% formic acid. The flow rate was set to 200 nL/min. The spray voltage was set to 2.7 kV and the temperature of the heated capillary was set to 40 °C. The ion transfer tube temperature was set to 275 °C. The scan range was 400–1600 m/z. The HCD collision energy was set to 50%, appropriate for fragmentation of glycopeptide ions. Precursor and fragment detection were performed using an Orbitrap at a resolution MS^1^ = 100,000. MS^2^ = 30,000. The AGC target for MS^1^ = 4e^5^ and MS^2^ = 5e^4^ and injection time: MS^1^ = 50 ms MS^2^ = 54 ms.

Glycopeptide fragmentation data were extracted from the raw file using Byonic™ (Version 3.5) and Byologic™ software (Version 3.5; Protein Metrics Inc.). The glycopeptide fragmentation data were evaluated manually for each glycopeptide; the peptide was scored as true-positive when the correct b and y fragment ions were observed along with oxonium ions corresponding to the glycan identified. The MS data were searched using the Protein Metrics N-glycan library. The relative amounts of each glycan at each site as well as the unoccupied proportion were determined by comparing the extracted chromatographic areas for different glycotypes with an identical peptide sequence. All charge states for a single glycopeptide were summed. The precursor mass tolerance was set at 4 ppm and 10 ppm for fragments. A 1% false discovery rate (FDR) was applied. The relative amounts of each glycan at each site as well as the unoccupied proportion were determined by comparing the extracted ion chromatographic areas for different glycopeptides with an identical peptide sequence. Glycans were categorized according to the composition detected. HexNAc(2)Hex(9–5) was classified as M9 to M5. HexNAc(3)Hex(5–6)X was classified as Hybrid with HexNAc(3)Fuc(1)X classified as Fhybrid. Complex-type glycans were classified according to the number of processed antenna and fucosylation. If all of the following compositions have a fucose they are assigned into the FA categories. HexNAc(3)Hex(3–4)X is assigned as A1, HexNAc(4)X is A2/A1B, HexNAc(5)X is A3/A2B, and HexNAc(6)X is A4/A3B. As this fragmentation method does not provide linkage information compositional isomers are grouped, so for example a triantennary glycan contains HexNAc 5 but so does a biantennary glycans with a bisect. Any glycan containing at least one sialic acid was counted as sialylated.

### Surface Plasmon Resonance (SPR)

SARS-COV-2 S and ACE2 proteins were buffer exchanged in HBS P+ buffer (Cytiva/GE Healthcare). All analysis was performed using a Biacore T200 (Cytiva/GE Healthcare). After removing contaminants via a pulse of EDTA (350 mM) for 1 min at a flow rate of 30 μL/min, the chip was loaded with Ni^2+^ by injecting NiCl_2_ for 1 min at a flow rate of 10 μL/min. SARS-CoV2 S protein (50 μg/ml) was injected at 10 μL/min for 240 s. Control channels were not loaded with trimer. Control cycles were performed by flowing ACE2 over Ni^2+^-loaded NTA in the absence of trimer. The analyte was injected into the trimer sample and control channels at a flow rate of 50 μL/min. Serial dilutions ranging from 200 nM to 3.125 nM were performed in triplicate along with HBS P + buffer only as a control. Association was recorded for 300 s and dissociation for 600 s. After each cycle of interaction, the NTA-chip surface was regenerated with a pulse of EDTA (350 mM) for 1 min at a flow rate of 30 μL/min. A high flow rate of analyte solution (50 μL/min) was used to minimize mass-transport limitation. The resulting data were fit to a 1:1 binding model using Biacore Evaluation Software (GE Healthcare) and these fitted curves were used to calculate *K*_D_. Paired t tests were used where possible to determine significance and for remaining samples where a direct experimental pairing was not possible an unpaired t test was performed. All statistics and graph plotting was performed using GraphPad prism v8.1.

## CRediT authorship contribution statement

**Joel D. Allen:** Conceptualization, Investigation, Formal analysis, Writing - original draft, Writing - review & editing. **Yasunori Watanabe:** Conceptualization, Investigation, Formal analysis, Writing - original draft, Writing - review & editing. **Himanshi Chawla:** Investigation, Writing - review & editing. **Maddy L. Newby:** Investigation, Writing - review & editing. **Max Crispin:** Conceptualization, Funding acquisition, Supervision, Writing - original draft, Writing - review & editing.
