# Supplementary material for: Subtle Influence of ACE2 Glycan Processing on SARS-CoV-2 Recognition
Source: J Mol Biol. 2021 Feb 19;433(4):166762. doi: 10.1016/j.jmb.2020.166762 (PMC7744274; doi:10.1016/j.jmb.2020.166762)

**Supplementary material for:**

**Subtle influence of ACE2 glycan processing on SARS-CoV-2 recognition**

Joel D. Allen^1#^, Yasunori Watanabe^1,2,3#^, Himanshi Chawla^1^, Maddy L. Newby^1^, Max Crispin^1*^

^1^ School of Biological Sciences, University of Southampton, Southampton, SO17 1BJ, UK

^2^ Oxford Glycobiology Institute, Department of Biochemistry, University of Oxford, South Parks Road, Oxford, OX1 3QU, UK

^3^ Division of Structural Biology, University of Oxford, Wellcome Centre for Human Genetics, Oxford, OX3 7BN, UK

^#^ These authors contributed to this work equally.

^*^ To whom correspondence may be addressed. Email: max.crispin@soton.ac.uk

**Supplementary Table 1: Site-specific glycan analysis of ACE-2**

**
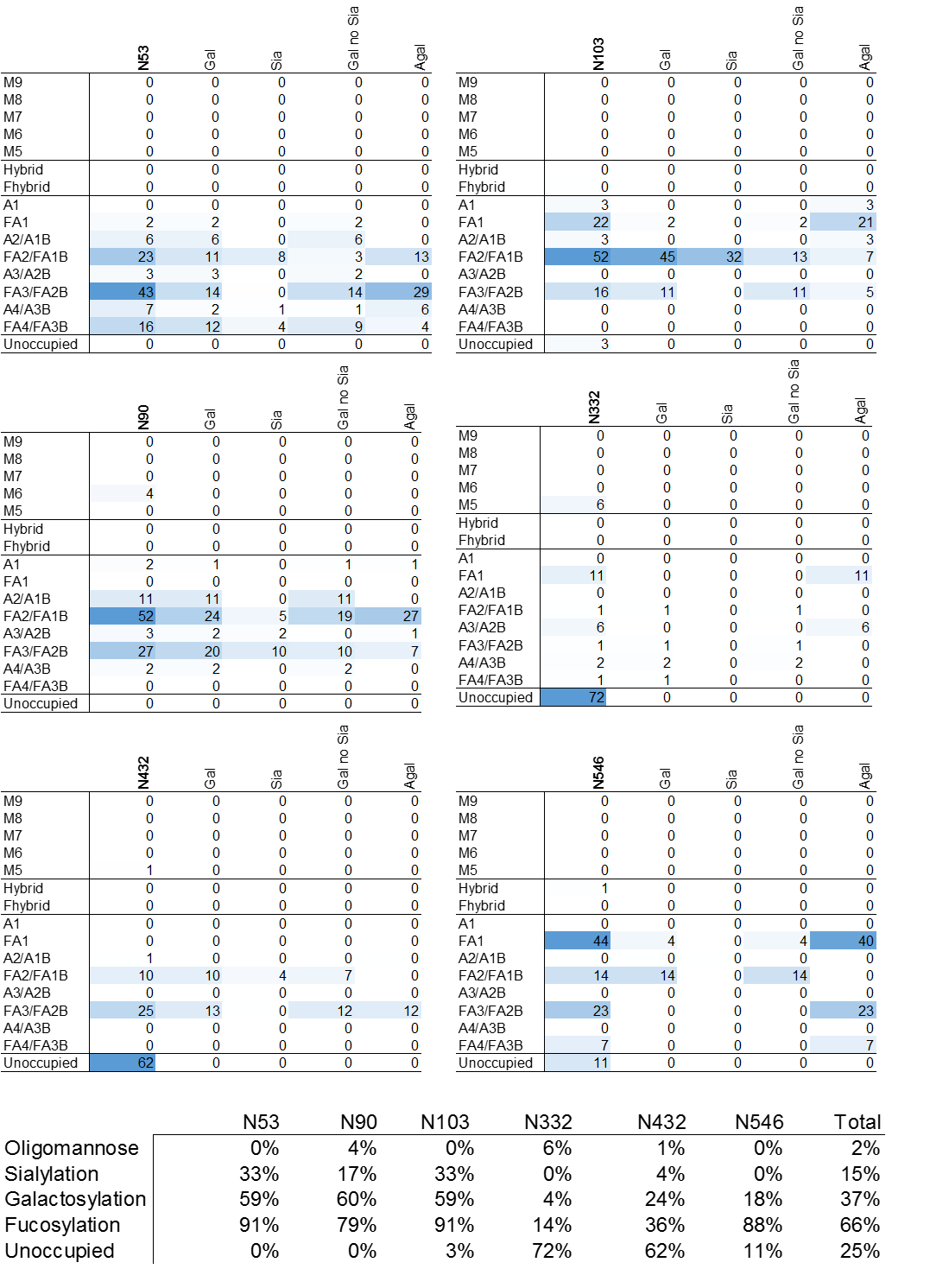
**

**Supplementary Table 2: Site-specific glycan analysis of Kif and ST6 ACE2**

**
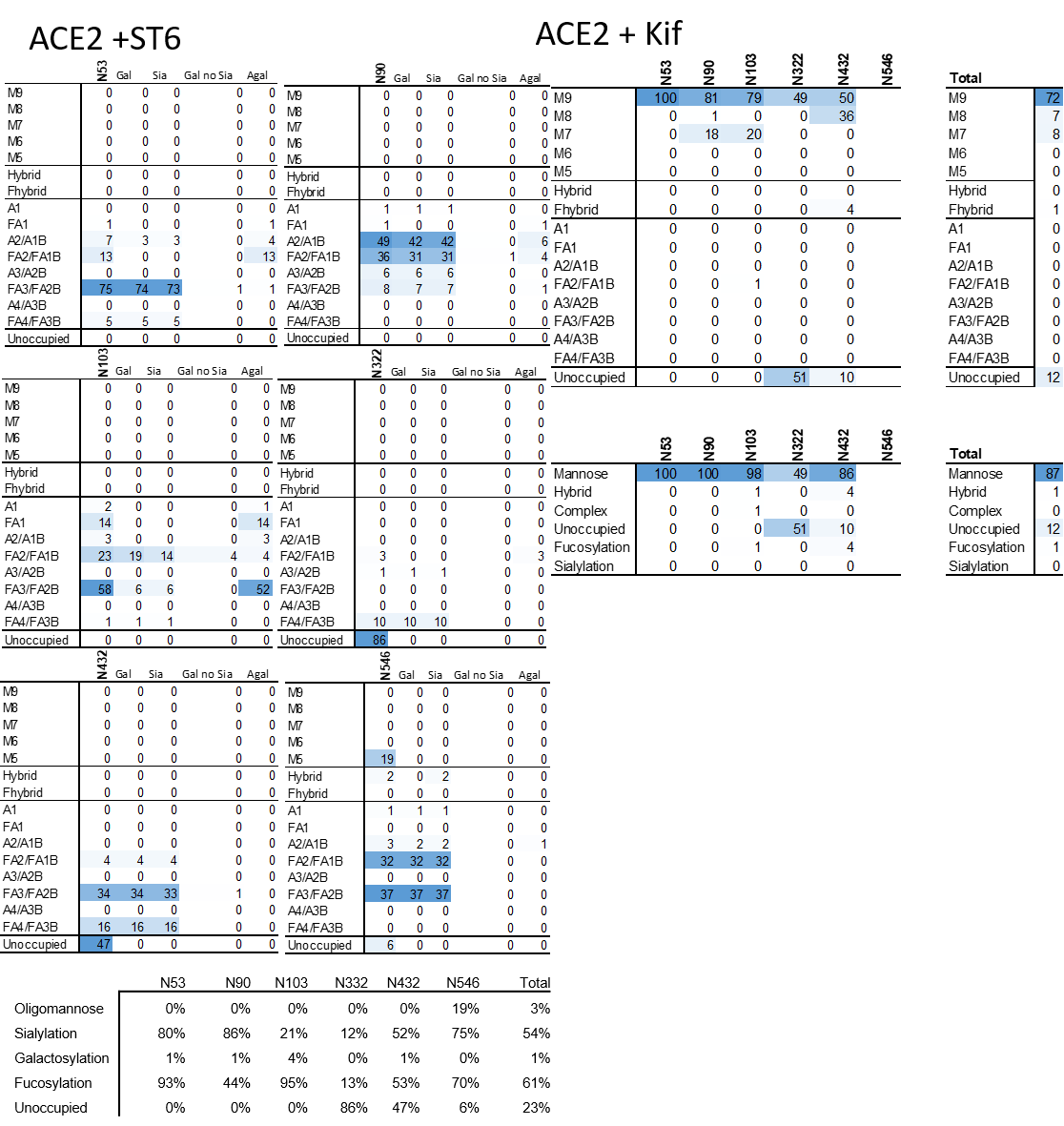
Supplementary Table 3: Detected glycopeptides for glycosidase treated ACE2 variants**

**
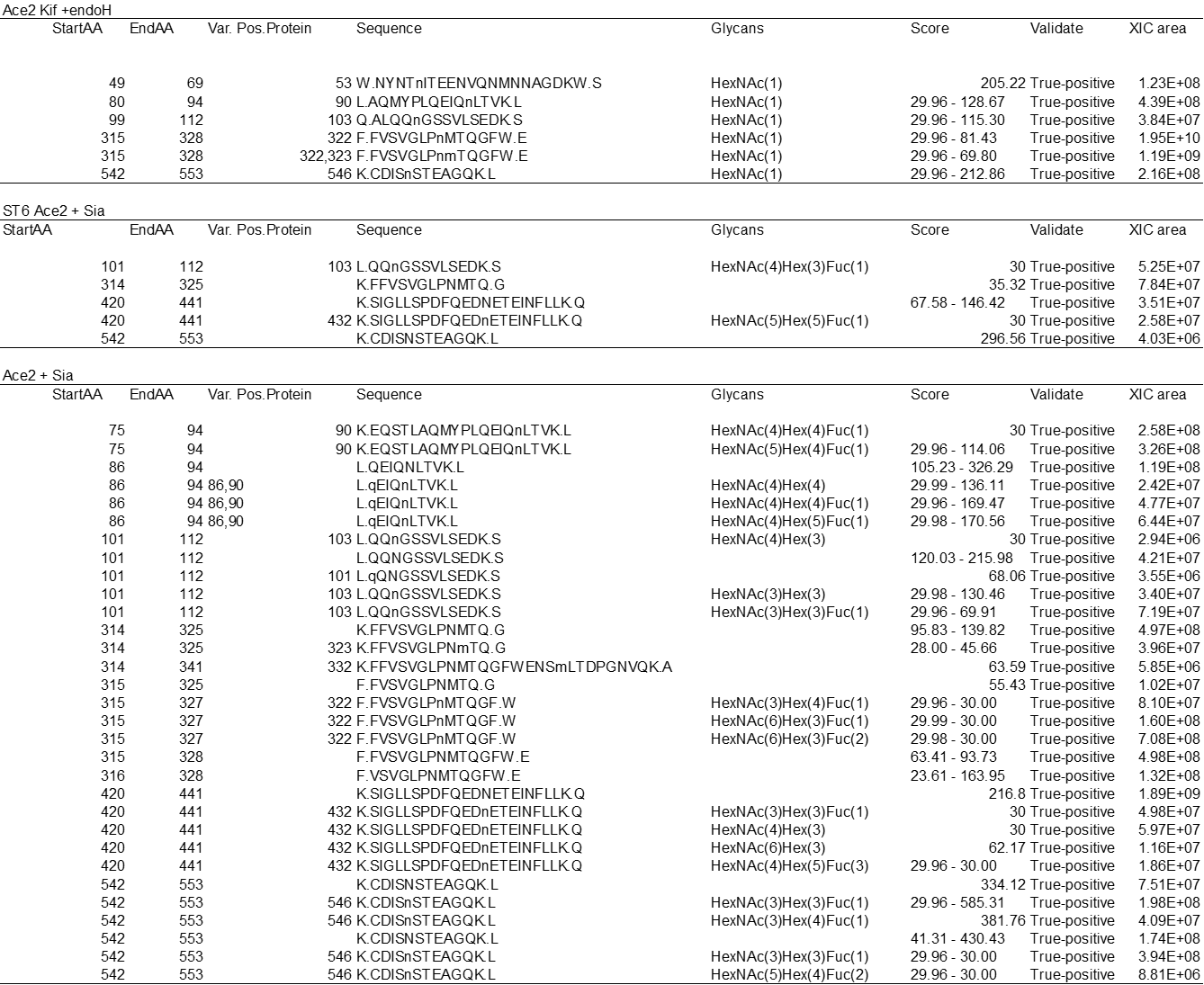
**

**Supplementary Table 4: Analysis of glycan engineered ACE2 SPR replicates**


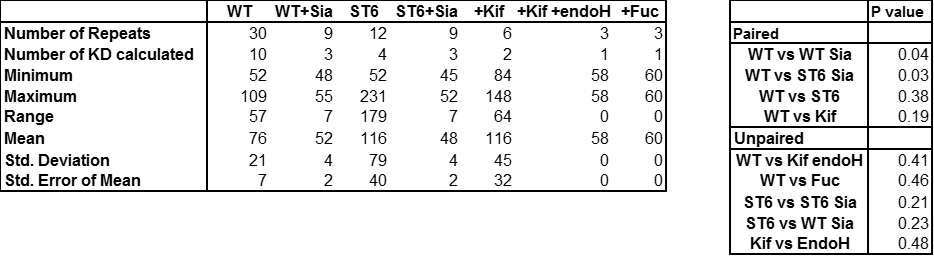

Supplement: Supplementary data 1 [file mmc1.docx]
